# Compared characteristics of current vs. past smokers at the time of diagnosis of a first-time lung or head and neck cancer: a cross-sectional study

**DOI:** 10.1186/s12885-018-4253-5

**Published:** 2018-04-03

**Authors:** Corinne Vannimenus, Hélène Bricout, Olivier Le Rouzic, François Mouawad, Dominique Chevalier, Eric Dansin, Laurence Rotsaert, Gautier Lefebvre, Olivier Cottencin, Henri Porte, Arnaud Scherpereel, Asmaa El Fahsi, Florence Richard, Benjamin Rolland, The ALTAK Study Group, Corinne Vannimenus, Françoise Weingertner, Hélène Bricout, Olivier Le Rouzic, François Mouawad, Dominique Chevalier, Eric Dansin, Stéphanie Clisant, Laurence Rotsaert, Gautier Lefebvre, Olivier Cottencin, Henri Porte, Arnaud Scherpereel, Asmaa El Fahsi, Dienabou Sylla, Florence RICHARD, Benjamin Rolland

**Affiliations:** 1 Service de Tabacologie, Clinique de Pneumologie, Hôpital Calmette, CHRU de Lille CS70001, 59037 Lille cedex, France; 2 Centre de Référence Régionale en Cancérologie, Lille, France; 3 Service d’Oto-rhino-laryngologie, CHRU de Lille, Lille, France; 4 Département de Cancérologie Cervico-Faciale, Centre de Lutte Contre le Cancer Oscar Lambret, Lille, France; 5 Service d’Addictologie, CHRU de Lille, Lille, France; 6 Clinique de Chirurgie Thoracique, CHRU de Lille, Lille, France; 7 Santé Publique et Epidémiologie, Institut Pasteur, Université de Lille, INSERM UMR744, Lille, France; 8 Univ Lyon; UCBL; INSERM U1028 ; CNRS UMR5292 ; Service Universitaire d’Addictologie de Lyon, CH le Vinatier, Lyon, France

**Keywords:** Tobacco use, Lung neoplasms, Head & Neck Neoplasms, Alcohol-related disorders

## Abstract

**Background:**

Active smoking at the time of diagnosis of a first head & neck (H&N) or lung cancer is associated with a worse cancer outcome and increased mortality. However, the compared characteristics of active vs. former smokers at cancer diagnosis are poorly known.

**Methods:**

In 371 subjects with a first H&N or lung cancer, we assessed: 1) socio-demographic features; 2) lifelong types of smoking; 3) alcohol use disorder identification test (AUDIT); 4) cannabis abuse screening test (CAST); and 5) Mini International Neuropsychiatric Interview (MINI). Using a multivariable regression model, we compared the profile of current smokers and past smokers.

**Results:**

Current smokers more frequently exhibited H&N cancer (OR 3.91; 95% CI [2.00–6.51]; *p* <  0.0001) and ever smoking of hand-rolled cigarettes (OR 2.2; 95% CI [1.25–3.88]; *p* = 0.007). Among subjects with lung cancer (*n* = 177), current smoking was primarily associated with ever smoking of hand-rolled cigarettes (OR 2.88; 95% CI [1.32–6.30]; *p* = 0.008) and negatively associated with age (OR 0.92; 95% CI [0.89–0.96]; *p* <  0.001). Among subjects with H&N cancer (*n* = 163), current smokers exhibited a significantly greater AUDIT score (OR = 1.08; 95% CI [1.01–1.16]; *p* = 0.03).

**Conclusion:**

At the time of diagnosis of the first lung or H&N cancer, current smoking is highly associated with previous type of smoking and alcohol drinking patterns.

**Trial registration:**

NCT01647425; Registration date: July 23, 2012.

## Background

Tobacco smoking is the most important risk factor for lung cancer [1], while tobacco and alcohol conjointly account for the occurrence of 67–84% of the head and neck (H&N) cancers [2]. Continuing to smoke after the diagnosis of cancer is associated with higher risks of complications, secondary cancers, and death [3]. Moreover, in patients with a lung or H&N cancer, persistent smoking is associated with decreased quality of life, general health, and emotional and social functioning [4–6]. Intensive smoking cessation programs are thus warranted in current smokers with a first tobacco-related cancer [7].

In recent years, the implementation of tobacco control policies has enhanced the level of information on tobacco-related harm, which has promoted tobacco cessation [8], and subsequently reduced the incidence of tobacco-related cancers [9]. Despite the impact of such policies, some individuals continue to smoke tobacco until they experience a first-time tobacco-related health problem, including a first-time tobacco-related cancer. In contrast, some other individuals who experience a tobacco-related cancer had already stopped tobacco use long before the cancer diagnosis. Thus, the profile of patients with a first-time tobacco-related cancer can be divided into three categories at cancer diagnosis: 1) current smokers (CSs); 2) former smokers (FSs); and 3) never smokers.

Many previous studies have compared the profile of current vs. former smokers at the time of cancer diagnosis. A comprehensive review has recently listed these studies [10]. However, these studies essentially consisted of unadjusted analyses on the smoking status at cancer diagnosis, and, in most cases, they did not explore the contribution of the psychiatric history and other substance use patterns. Consequently, little is known about whether the smoking status at the time of diagnosis of a first-time lung or H&N cancer is related to specific social or psychiatric features, lifelong smoking patterns, or history of other substance uses. In the non-cancer population, it was found that the overall outcome of tobacco dependence is associated with the age of first cigarette, lifelong smoking patterns, and poorer psychosocial conditions [11]. Moreover, concurrent psychiatric and other substance use disorders are also associated with a poorer outcome [12, 13]. In patients with a first lung or H&N cancer, the role of these risk factors was never investigated. Enhancing the knowledge of the psychosocial determinants of the CS status among subjects with a first lung or H&N cancer could strengthen the impact of smoking cessation programs proposed to these patients.

In a multicenter one-year cohort study among 371 subjects with a first-time lung or H&N cancer (i.e., the ALTAK study), we conducted a cross-sectional study using the baseline assessment to investigate the differences in social factors, psychiatric condition, and other substance use patterns between current and past-smokers at the time of the cancer diagnosis. The presentation of the study is provided according to the ‘strengthening the reporting of observational studies in epidemiology’ (STROBE) statement [14].

## Methods

### Study design and centers

Data used in this study originated from the baseline screening of the ALTAK cohort study, conducted among 372 subjects with a first lung or H&N cancer. Participants were recruited by otorhinolaryngologists, clinical oncologists, or pulmonologists of the participating centers, at the end of the consultation of cancer announcement. Physicians explained the study and received written consent for participation. The study took place between September 2012 and December 2014 in three different centers: 1) the *Centre Oscar Lambret,* i.e., the Regional Comprehensive Cancer Center of Lille; 2) the Department of Respiratory Diseases of the University Hospital of Lille; and 3) the Department of Otolaryngology-Head and Neck Surgery of the University Hospital of Lille.

The main objectives of the ALTAK cohort study were: 1) to measure the proportion of subjects that maintain tobacco smoking or alcohol abuse despite the occurrence of cancer; and 2) to identify the social and psychiatric features and addictive comorbidities that could constitute vulnerability factors for not stopping tobacco or alcohol after cancer diagnosis. More information on the study protocol can be found at https://clinicaltrials.gov/ct2/show/NCT01647425. Here, we have used the baseline data of the cohort to explore the determinants of the smoking status at time of cancer diagnosis.

### Participants and measures

Inclusion criteria were: 1) age of 18 or more; 2) first lung or H&N cancer excluding mesothelioma and esophagus cancer; and 3) no history of any other cancer over the last five years. The type of cancer (lung or H&N), and the TNM classification grade [15] were noted by the clinician who received the initial consent.

Participants were assessed by an addiction specialist in the week following the announcement of the cancer diagnosis. The information collected at baseline and used in the present study was: 1) socioeconomic conditions: age, gender, familial status, employment, and educational level; 2) cancer status: localization, TNM grade, Eastern Cooperative Oncology Group / World Health Organization (ECOG/WHO) score; 3) smoking status and smoking habits: CS, i.e., at last one smoking episode in previous month; FS, i.e., no smoking episode over the previous month; and lifelong non-smoker; 4) alcohol use patterns: use of alcohol during the last 12 months (yes/no); misuse of alcohol during the last 12 months (yes/no), defined by an average alcohol use exceeding the recommended national thresholds, i.e., ≥210 g of alcohol per week for a man and 140 g for a woman, and ≥ 50 g per occasion for a man and 40 g for a woman [16], Alcohol Use Disorder Identification Test (AUDIT) [17]; 5) Severity of use of cannabis using the Cannabis Abuse Screening Test (CAST) [18], i.e., at least one positive item; and 6) Psychiatric assessment using the Mini International Neuropsychiatric Interview (MINI) [19], which is a validated structured interview for diagnosing mental health disorders.

### Statistical analyses

Descriptive statistics of each variable are reported in Table 1. Quantitative values are presented as the mean (standard deviation [sd]) when normally distributed or as the median (interquartile range) when there is skewed distribution. Qualitative data are presented as n (percent). To compare smoker and ex-smoker groups, Student’s t-test or the Kruskal-Wallis test were used for quantitative data, and the Chi-squared test or Fisher’s exact test were used for categorical data. Logistic regression models were used to estimate OR with 95% confidence interval (95%CI). A backward stepwise regression method was used to select variables (with *p* < 0.20) associated with smoking status in a multiple logistic regression model, adjusted for age and sex. Significance levels were set at *p* < 0.05. The final multiple logistic regression model was also stratified by localization. Analyses were performed using the SAS software release 9.02 (SAS Institute INC, Cary, NC, USA).

**Table 1:** Descriptive statistics and univariate comparisons between current and past smokers at the time of the cancer onset

	Past Smokers	Current Smokers	*p*-value
Number of subjects (n; %)	177 (52.1%)	163 (47.9%)	
Gender (n females; %)	27 (15.3%)	35 (21.5%)	0.14
Age (m ± SD)	61.9 ± 9.7	56.0 ± 8.0	< 0.0001
Educational level			0.45
Elementary / primary school	75 (42.4%)	68 (41.7%)	
Secondary / high school	75 (42.4%)	77 (47.2%)	
Undergraduate and more/ college or university	27 (15.2%)	18 (11.1%)	
Workers (n; %)	58 (32.8%)	98 (60.1%)	< 0.0001
Married (n; %)	124 (70.1%)	97 (59.5%)	0.04
Living alone (n; %)	35 (19.8%)	45 (27.6%)	0.09
Cancer			
Localization			< 0.0001
1 = lung	119 (67.2%)	58 (35.6%)	
2 = head & neck	58 (32.8%)	105 (64.4%)	
“0” score at the ECOG performance status (n; %)	77 (43.5%)	47 (28.8%)	0.005
TNM Grade 4 (*N* = 299) (n; %)	97 (59.2%)	73 (54.1%)	0.38
Presence of metastases (*N* = 296) (n; %)	72 (44.7%)	40 (29.6%)	0.008
Tobacco			
Age of first cigarette (years; m ± SD)	15.6 ± 3.8	15.6 ± 4.1	0.95
Lifelong reported types of smoking, (*N* = 339) (n; %)			
Manufactured cigarettes	172 (97.2%)	154 (95.1%)	0.31
Roll-up cigarettes	64 (36.2%)	91 (56.2%)	0.0002
Cigarillos	82 (46.3%)	58 (35.8%)	0.05
Cigars	55 (31.1%)	40 (24.7%)	0.19
Pipe	50 (28.3%)	26 (16.1%)	0.007
Alcohol			
12-month use (n; %)	140 (79.1%)	122 (74.8%)	0.35
12-month misuse (n; %)	51 (28.8%)	70 (42.9%)	0.007
AUDIT, (N = 322) (median [IQ])	4 [2–6]	6 [2–11]	0.002^a^
AUDIT-C, (*N* = 322) (median [IQ])	4 [1–5]	5 [1–7]	0.01^a^
CAST (m ± SD)	0.10 ± 0.43	0.22 ± 0.59	0.019^a^
MINI 5.0 (*N* = 337)			
TOTAL (n; %)	61 (34.9%)	72 (44.4%)	0.07
Current MDD (n; %)	23 (13.1%)	29 (17.9%)	0.22
Dysthymia (n; %)	2 (1.1%)	3 (1.9%)	0.67^b^
Suicide risk (n; %)	39 (22.3%)	43 (26.5%)	0.36
Lifelong mania/hypomania (n; %)	2 (1.1%)	7 (4.3%)	0.09^b^
Schizophrenia (n; %)	10 (5.7%)	14 (8.6%)	0.30
Panic disorder/ agoraphobia (n; %)	16 (9.1%)	27 (16.7%)	0.04
Eating Disorder (n; %)	0 (0.0%)	0 (0.0%)	NA
Generalized anxiety (n; %)	7 (4.0%)	3 (1.9%)	0.34^b^
Antisocial personality disorder (n; %)	0 (0.0%)	3 (1.9%)	0.12^b^
Obsessive-compulsive disorder (n; %)	1 (0.6%)	2 (1.2%)	0.61^b^
PTSD (n; %)	1 (0.6%)	4 (2.5%)	0.20^b^
Other SUD (n; %)	0 (0.0%)	1 (0.6%)	0.48^b^

Abbreviations: *AUDIT*: Alcohol Use Disorder Identification Test, AUDIT-C: AUDIT “consumptions” i.e., the 3 first questions of the AUDIT, which pertain to the drinking levels, *CAST*: Cannabis Abuse Screening Test, *ECOG*: Eastern Cooperative Oncology Group, *MINI*: Mini International Neuropsychiatric Interview, version 5.0, *TNM*: Tumor/Nodules/Metastases, *PTSD*: Post-Traumatic Stress Disorder, *SUD*: Substance Use Disorder

*a “0” score at the ECOG performance status means “Fully active, able to carry on all predisease activities without restriction”

^a^Mann Whitney test

^b^Fischer’s exact test

### Ethics approval

The protocol of the ALTAK study (NCT01647425) was declared to and approved by the Comité de Protection des Personnes Nord-Ouest (#CPP12/09) and the Agence Nationale des Médicaments et produits de santé (#B111675–10).

## Results

In total, 389 subjects were proposed to participate in the ALTAK study, of whom 371 accepted (response rate: 95.4%). Among them, 163 (43.9%) were CSs at the time of cancer diagnosis, and 177 (47.7%) were PSs. The characteristics of these participants are summarized in Table 1, according to their smoking status. Bivariable comparisons between the CS and FS groups are also provided in Table 1.

Bivariable comparisons found that belonging to the CS group was significantly associated with younger age (*p* < 0.0001), being professionally active (p < 0.0001), and being unmarried (*p* = 0.04). Moreover, the CS status was significantly associated with a H&N localization of the cancer, with no alteration in daily life activities at the time of diagnosis (*p* = 0.005), and with the absence of metastases (*p* = 0.008). With respect to recent substance use patterns, being CS was associated with smoking roll-up cigarettes (*p* < 0.0002), with reporting alcohol misuse in the past 12 months (*p* = 0.007) as well as with the AUDIT (*p* = 0.002) and AUDIT-C (*p* = 0.01) scores. Finally, the CS status was significantly associated with the CAST score for cannabis use (*p* = 0.019). By contrast, the CS status was not found associated with sex (*p* = 0.14), educational level (*p* = 0.45), or living alone (*p* = 0.09). Furthermore, being CS was not related to the age of first cigarette (*p* = 0.95) and the presence of any psychiatric disorder according to the MINI (*p* = 0.07).

A separate multivariable analysis of the CS status was conducted among patients with lung and H&N cancer (see Table 2). In subjects with a lung cancer, the CS status was positively associated with ever use of hand-rolled cigarettes and negatively associated with age and ever use of cigarillos. There were trends for positive statistical associations between the CS status and positive MINI, never use of a pipe, and being single. In subjects with H&N cancer, the CS status was positively associated with the AUDIT score. Trends for positive statistical associations were found between the CS status and younger age, ever use of hand-rolled cigarettes, and never use of a pipe.

**Table 2:** Association between patient characteristics and current smoking status. Logistic regression model adjusted for age and sex and stratified by localization

	Lung cancer			Head & neck cancer		
	OR	95% CI	*p*-value	OR	95% CI	*p*-value
Age	0.92	0.89–0.96	< 0.001	0.97	0.92–1.01	0.15
Marital status						
Married or in couple	1			1		
Single or widowed	0.49	0.23–1.07	0.07	0.70	0.30–1.61	0.40
Hand-rolled cigarettes						
Never use	1			1		
Ever use	2.88	1.32–6.30	0.008	1.98	0.93–4.25	0.08
Cigarillos						
Never use	1			1		
Ever use	0.43	0.19–0.97	0.04	1.09	0.46–2.58	0.85
Pipe						
Never use	1			1		
Ever use	0.46	0.16–1.37	0.16	0.37	0.14–1.00	0.05
AUDIT	1.01	0.92–1.10	0.87	1.08	1.01–1.16	0.03
Psychiatric disorder						
No	1			1		
Yes	1.89	0.86–4.13	0.11	1.36	0.60–3.11	0.46

AUDIT = Alcohol use disorders identification test

## Discussion

The main objective of the study was to compare the sociodemographic features, psychiatric history, and substance use patterns, of CSs and FSs at the time of initial diagnosis of a first lung or H&N cancer. The main results of the multivariable regression models were that the CS status was associated with younger age and ever use of hand-roll cigarettes in subjects with a first lung cancer, and increased AUDIT score in subjects with a first H&N cancer. Overall, we found a prevalence of 43.9% CSs, i.e., smokers over the preceding month. This figure is relatively consistent with the estimation provided by a recent literature review, which estimated approximately 50% the rate of CSs over the year preceding the diagnosis of a lung or H&N cancer [10]. Though most of the studies have used a one-year period prior to cancer to define the CS status [10], we chose to follow the recommendations of the National Cancer Institute that deem a one-month period to be more precise [20].

Regarding the sociodemographic features of CSs, we have found in bivariable comparisons that being CS was significantly associated with younger age, being inactive, and being unmarried. Previous studies that explored the smoking status among subjects with a first lung or H&N cancer found relatively similar results. For example, Schnoll et al. found that only a single marital status was significantly associated with increased CS status [21]. In subjects with H&N cancer only, Duffy et al. found that smoking at cancer diagnosis was associated with younger age, single status, and lower education level [22]. Another study by the same team of authors also found that active smoking at the time of diagnosis of an H&N cancer was associated with a single status, but not with age or education level [23].

Though younger age was not consistently found as a substantial contributor of the CS status in previous studies among subjects with lung or H&N cancer, it is a well-known factor in the general population, both in the US and in Europe [24, 25]. In this respective, our result could thus be reflective of this general finding. Moreover, the CS status was significantly more important among subjects with a H&N cancer, compared to those with a lung cancer.

Another important result was that the CS rate was much higher in the subjects with a first H&N cancer than in those with a first lung cancer. To our knowledge, our study was the first to directly compare these two types of populations. The risk of experiencing a lung cancer, especially adenocarcinoma, is still significantly increased compared with never smokers more than 30 years after smoking cessation [26]. In contrast, quitting tobacco smoking fosters a more rapid decrease in the relative risk of H&N cancer [27]. The gap observed in the CS rates between lung and H&N cancer populations may thus be attributable to this difference in longitudinal risk reduction among FSs.

More importantly, it was recently noted that almost no previous studies have addressed the relative rates of the types of tobacco ever used among CSs and FSs with a lung or H&N cancer and that it was a significant issue to explore [10]. In this regard, our study is also the first to provide important findings on this subject. We have notably found that reporting ever smoking of roll-up cigarettes was highly associated with being a CS at the time of cancer diagnosis of a first lung cancer. In the French population, it has been previously found that 24.3% of smokers were used handrolling tobacco, while 7.5% of smokers use only this type of tobacco [28]. These figures are difficult to compare to other countries, as the prevalence of roll-you-own smokers is very variable depending on countries and their specific regulations on tobacco [29]. Regardless, in France, compared to smokers of manufactured cigarettes, handrolling smokers reported much lower average personal incomes and higher rates of unemployment [28]. This finding is consistent with other data out of France [30]. In the general population, it was previously shown that quitting smoking is inversely associated with impaired social conditions (10). Our findings in patients with cancer could thus be explained by unexplored social factors, even if our analyses were adjusted for the level of education.

Moreover, as early alterations in daily life activities and the presence of metastases at the time of cancer diagnosis were significantly associated with the FS status in bivariable comparisons, it could be suggested that the FS status is actually partially explained by symptom burden, in particular the fact that some people have become too sick to smoke before cancer was diagnosed. However, these two variables were involved in the step-by-step regression model, but they were not retained as relevant explanatory parameters by the modeling.

Finally, we identified that concurrent alcohol misuse, reflected by the AUDIT score, was significantly associated with the CS status only in patients with a H&N cancer (see Table 2), which could be underlain by the fact that alcohol and tobacco are combined risk factors for this type of cancer.

Several limitations should be acknowledged with regard to the present study. First, the study was multicenter, but the recruitment was regional. Some findings could thus be skewed by local features. Moreover, patients with esophageal cancers were not included in the study, and thus, the findings cannot be applied to all types of H&N cancers. Finally, we did not assess the use of non-smoking types of tobacco, notably chewing or snuff tobacco. However, this type of tobacco use is very rare in Europe [25]. The main objective of our study was to assess the lifelong types of tobacco used by the patients. This retrospective assessment could be found to be rather imprecise. However, a detailed and quantitative assessment of the specific periods of time during which each type of tobacco was used over the entire lifetime period would have been very difficult to carry out and would have suffered from memory bias and imprecision.

## Conclusions

The study highlights important risk factors associated with a CS status at the time of diagnosis of a first lung or H&N cancer. Some risk factors are specific to H&N cancer (i.e., concurrent alcohol misuse), whereas some others are associated with both types of cancer, i.e., young age, ever use of hand-rolled cigarettes, and possibly some psychiatric comorbidities. These findings should warrant a specific screening of these risk factors in subjects with a first lung or H&N cancer, with the aim to treat comorbid conditions and to act on impaired social situations, together with treating cancer and offering tobacco cessation programs to the patients.

## Abbreviations

AUDIT: Alcohol Use Disorders Identification Test
CAST: Cannabis Abuse Screening Test
CSs: Current Smokers
ECOG: Eastern Cooperative Oncology Group
FSs: Former Smokers
H&N: Head and Neck
MINI: Mini International Neuropsychiatric Interview
STROBE: ‘Strengthening The Reporting of OBservational studies in Epidemiology’
WHO: World Health Organization
